# Recent Developments and Applications of the MMPBSA Method

**DOI:** 10.3389/fmolb.2017.00087

**Published:** 2018-01-10

**Authors:** Changhao Wang, D'Artagnan Greene, Li Xiao, Ruxi Qi, Ray Luo

**Affiliations:** ^1^Chemical and Materials Physics Graduate Program, University of California, Irvine, Irvine, CA, United States; ^2^Department of Molecular Biology and Biochemistry, University of California, Irvine, Irvine, CA, United States; ^3^Department of Physics and Astronomy, University of California, Irvine, Irvine, CA, United States; ^4^Department of Biomedical Engineering, University of California, Irvine, Irvine, CA, United States; ^5^Department of Chemical Engineering and Materials Science, University of California, Irvine, Irvine, CA, United States

**Keywords:** molecular recognition, binding affinity, free energy simulation, MMPBSA, continuum solvation model

## Abstract

The Molecular Mechanics Poisson-Boltzmann Surface Area (MMPBSA) approach has been widely applied as an efficient and reliable free energy simulation method to model molecular recognition, such as for protein-ligand binding interactions. In this review, we focus on recent developments and applications of the MMPBSA method. The methodology review covers solvation terms, the entropy term, extensions to membrane proteins and high-speed screening, and new automation toolkits. Recent applications in various important biomedical and chemical fields are also reviewed. We conclude with a few future directions aimed at making MMPBSA a more robust and efficient method.

## Introduction

It is widely accepted that high-level quantum mechanical (QM) methods provide the most detailed and accurate description of molecular structures, dynamics, and functions. However, for many biochemical systems that are often too complex, and/or biochemical processes that are too long, classical approaches are more commonly employed due to their efficiency and reasonable accuracy. To model biochemical systems classically, both long-range polar and short-range non-polar interactions are important for accurate and transferrable models (Perutz, 1978; Davis and McCammon, 1990; Honig and Nicholls, 1995). Among the classical simulation methods, the Molecular Mechanics Poisson-Boltzmann Surface Area (MMPBSA) approach (Srinivasan et al., 1998; Kollman et al., 2000; Gohlke and Case, 2004; Yang et al., 2011; Miller et al., 2012; Wang C. H. et al., 2016) has emerged as an efficient and reliable method to model molecular recognition, such as for protein-ligand binding interactions.

The development of binding free energy calculation methods has been a central focus in molecular simulations. Indeed, theoretically rigorous, but computationally expensive, free energy perturbation, and thermodynamic integration methods were both proposed much earlier than the MMPBSA method (Zwanzig, 1954; Bennett, 1976; Straatsma and McCammon, 1991). For simple and small systems where they can be reliably applied, these “exact” methods have been shown to be more accurate than the MMPBSA method. However, their applications to typically large and complex biomolecular recognition problems are quite limited. This is due to low efficiency and slow convergence. There are several key approximations utilized in the MMPBSA method that allow it to be used as an efficient and reasonable approximation for free energy simulations. The PBSA model is used so that the solvation contribution to the free energy is approximated by using a continuum solvent model. In addition, the method separately approximates enthalpy and entropy contributions to the free energy, with varying degrees of success as discussed below. There are other approximate methods that are similar to MMPBSA, such as the Mining Minima (M2) method and the Linear Interaction Energy (LIE) method. These are all categorized as end-point methods that focus on the end states of processes to compute the free energy changes (Head et al., 1997; Luo et al., 1999, 2001; Luo and Gilson, 2000; Aqvist and Marelius, 2001; Chen et al., 2004; Chang et al., 2007; Moghaddam et al., 2011; Mikulskis et al., 2012; Muddana and Gilson, 2012; Muddana et al., 2014).

In this review, we focus on recent developments and applications of the MMPBSA method that have been reported since 2014, which was roughly the time when the last major MMPBSA review was published (Genheden and Ryde, 2015). In the following, we first review the improvements made to the MMPBSA method over the last few years. This is followed by recent applications of the method in various fields, with a focus on biomedical applications.

## Improvements of MMPBSA

### Overview of MMPBSA

The MMPBSA method is most often applied to the calculation of binding free energies (ΔG_bind_) of small molecule ligands bound to large biomolecule receptors, although large inter-biomolecular recognitions are also often reported as reviewed below. The binding free energy of the bound ligand-receptor complex in an aqueous solvent (ΔG_bind, aq_) can be approximated as (Srinivasan et al., 1998; Kollman et al., 2000):

(1) $$\Delta {\text{G}}_{\text{bind},\text{aq}}=\Delta \text{H}-\text{T}\Delta \text{S}\approx \Delta {\text{E}}_{\text{MM}}+\Delta {\text{G}}_{\text{bind},\text{solv}}-\text{T}\Delta \text{S},$$

(2) $$\Delta {\text{E}}_{\text{MM}}=\Delta {\text{E}}_{\text{covalent}}+\Delta {\text{E}}_{\text{electrostatic}}+\Delta {\text{E}}_{\text{vdW}},$$

(3) $$\Delta {\text{E}}_{\text{covalent}}=\Delta {\text{E}}_{\text{bond}}+\Delta {\text{E}}_{\text{angle}}+\Delta {\text{E}}_{\text{torsion}},$$

(4) $$\Delta {\text{G}}_{\text{bind},\text{solv}}=\Delta {\text{G}}_{\text{polar}}+\Delta {\text{G}}_{\text{non-polar}},$$

where ΔE_MM_, ΔG_bind, solv_, and − TΔS represent the gas-phase molecular mechanical energy change, the solvation free energy change, and the conformational entropy change upon binding, respectively. All of these changes are computed via ensemble averaging over a large set of sampled conformations. ΔE_MM_ includes three terms calculated using molecular mechanics (MM): the covalent energy change (ΔE_covalent_), the electrostatic energy change (ΔE_electrostatic_), and the van der Waals energy change (ΔE_vdW_). ΔE_covalent_ consists of changes in the bond terms (ΔE_bond_), the angle terms (ΔE_angle_), and the torsion terms (ΔE_torsion_). The solvation free energy change (ΔG_bind, solv_) is usually separated into polar and non-polar contributions (ΔG_polar_ and ΔG_non-*polar*_). The entropy term is the most difficult to compute, and it is often approximated with a normal mode method using a few selected snapshots.

To compute all the ensemble averages in Equations (1–4) for a binding affinity calculation, the MMPBSA method usually begins with a molecular dynamics (MD) simulation of the complex using the single-trajectory approach, or three separate MD simulations of the complex, receptor, and ligand, respectively, in the multi-trajectory approach. A snapshot of a structure is taken at various time points during the production portion of the MD simulations. These snapshots are then used to calculate average values and uncertainties of various quantities of interest. The MD simulations are almost always conducted in an explicit solvent model to obtain the most accurate snapshots possible before carrying out any calculations that make use of them. It is important to obtain many different conformations, or snapshots, over a suitable timeframe for use in later statistical analysis as it is often not trivial to observe converged averaging, even in the relatively easier single-trajectory approach (Wang C. H. et al., 2016).

### The polar solvation term

For the calculation of the solvation free energy change (ΔG_bind, solv_), the explicit solvent is removed and replaced with an implicit continuum solvent to greatly speed up the calculation time. The polar solvation term (ΔG_polar_) in Equation (4) is calculated using a finite-difference solution or a Generalized Born (GB) pairwise approximation of the Poisson-Boltzmann equation (PBE) (Warwicker and Watson, 1982; Bashford and Karplus, 1990; Jeancharles et al., 1991; Gilson, 1995; Edinger et al., 1997; Luo et al., 1997, 2002; Lu and Luo, 2003; Tan et al., 2006; Cai et al., 2009, 2010, 2011; Wang et al., 2009, 2010, 2012, 2013; Ye et al., 2009, 2010; Wang and Luo, 2010; Hsieh and Luo, 2011; Botello-Smith et al., 2012; Liu et al., 2013; Wang C. H. et al., 2017). In addition to modeling binding interactions, PBE methods have also been applied to the prediction of pKa values for ionizable groups in biomolecules (Luo et al., 1998; Georgescu et al., 2002; Nielsen and McCammon, 2003; Warwicker, 2004; Tang et al., 2007), solvation free energies (Nicholls et al., 2008; Shivakumar et al., 2009), and protein folding and design (Hsieh and Luo, 2004; Wen and Luo, 2004; Wen et al., 2004; Lwin and Luo, 2005, 2006; Marshall et al., 2005; Lwin et al., 2006; Korman et al., 2008; Tan and Luo, 2008, 2009).

The PBE is based on the more fundamental Poisson equation:

(5) $$\begin{array}{r} \nabla \cdot \varepsilon \left(\vec{r}\right)\nabla \phi \left(\vec{r}\right)=-4\pi \rho^{f}\left(\vec{r}\right), \end{array}$$

where $\varepsilon \left(\vec{r}\right)$ is a predefined dielectric distribution function for the solvated molecular system, $\phi \left(\vec{r}\right)$ is the potential distribution function, and $\rho^{f}\left(\vec{r}\right)$ is the fixed atomic charge density. To model the electrostatic interaction due to the additional presence of salt in the aqueous solution, the electrostatic potential $\phi \left(\vec{r}\right)$ can be described by the PBE as:

(6) $$\nabla \cdot \varepsilon \left(\vec{r}\right)\nabla \phi \left(\vec{r}\right)+\lambda \left(\vec{r}\right)f\left(\phi \left(\vec{r}\right)\right)=-4\pi \rho^{f}\left(\vec{r}\right)\;,$$

with

(7) $$f\left(\phi \left(\vec{r}\right)\right)=4\pi \sum_{i}^{N}z_{i}ec_{i}\exp\left(-\frac{z_{i}e\phi \left(\vec{r}\right)}{k_{B}T}\right).$$

Here, $\lambda \left(\vec{r}\right)$ is a predefined ion-exclusion function with a value of 0 within the Stern layer and the molecular interior and a value of 1 outside the Stern layer. The salt-related term $f\left(\phi \left(\vec{r}\right)\right)$ is a function of the potential, $\phi \left(\vec{r}\right),$ the valence, *z*_*i*_, of ion type *i*, and the bulk concentration, *c*_*i*_, at a given temperature *T*. When both the ionic strength and electric field are weak, the PBE can be linearized for easier numerical solutions:

(8) $$\nabla \cdot \varepsilon \left(\vec{r}\right)\nabla \phi \left(\vec{r}\right)=-4\pi \rho^{f}\left(\vec{r}\right)+\varepsilon_{\nu }\kappa^{2}\phi \left(\vec{r}\right),$$

where $\kappa^{2}=\frac{8\pi e^{2}I}{{\varepsilon_{v}k}_{B}T}$. Here ε_ν_ denotes the solvent dielectric constant, and *I* represents the ionic strength of the solution.

Over the past few years, a few new algorithm developments were reported for the numerical solution of the PBE (Xie, 2014; Fisicaro et al., 2016; Xie and Jiang, 2016). To deal with the singularity and nonlinearity of the PBE, Xie proposed a new decomposition and minimization scheme, together with a new proof on the existence and uniqueness of the PBE solution. A new PBE finite element solver was developed based on these solution decomposition and minimization techniques (Xie, 2014). Fisicaro et al. presented a preconditioned conjugate gradient technique to solve the generalized Poisson problem, and the linear regime of the PBE, in some 10 iterations. In combination with a self-consistent procedure, this technique was able to solve the non-linear Poisson–Boltzmann problem in a formulation including ionic steric effects (Fisicaro et al., 2016). Later Xie et al. incorporated nonlocal dielectric effects into the classic PBE for a protein in ionic solvent to derive a nonlocal modified Poisson–Boltzmann equation (NMPBE) and developed a finite element algorithm with a related package for solving the NMPBE (Xie and Jiang, 2016). Their results demonstrate the potential for the NMPBE to be a better predictor of electrostatic solvation and binding free energies compared to the standard PBE. It is worth noting that there has been a community wide push to explore alternative hardware for biomolecular simulations, such as the graphics processing units (GPU), which have a parallel architecture and are suited for high-performance computation with dense data parallelism (Colmenares et al., 2014a,b; Qi R. et al., 2017). A finite difference scheme with the successive over-relaxation method was implemented on the CUDA-based GPUs in the DelPhi package, which achieved a speedup of ~10 times in the linear and non-linear cases (Colmenares et al., 2014b). More recently, Qi et al. implemented and analyzed commonly used linear PBE solvers on CUDA GPUs for biomolecular simulations, including both standard and preconditioned conjugate gradient (CG) solvers with several alternative preconditioners (Qi R. et al., 2017). After extensive testing, the optimal GPU performance was observed using the Jacobi-preconditioned CG solver with a significant speedup that was up to 50 times faster than the standard CG solver on CPU. These progressive efforts on efficient numerical PBE solvers show great potential for accelerating MMPBSA computation.

Since the prior review (Genheden and Ryde, 2015), the numerical procedure and related factors for the widely used finite-difference method were also investigated for their impact on the MMPBSA method (Wang C. H. et al., 2016). This study showed that the impact of grid spacing on the quality of MMPBSA calculations is small in protein-ligand binding calculations; the agreement with experiment changed by a negligible amount when the grid spacing was changed from 0.50 to 0.25 Å. This indicated that the widely adopted default value of 0.50 Å used by the community was sufficient. The impact of different atomic radius sets and different molecular surface definitions was also analyzed, and weak influences were found on the agreement with experiment (Wang C. H. et al., 2016). This is probably due to the use of high protein dielectrics for the often-charged ligands and/or active sites as discussed below.

The effect of the solute dielectric constant was also investigated. A higher solute dielectric constant (using 2 or 4 instead of 1) was found to perform better in the virtual screening of ligands for tyrosine kinases (Sun et al., 2014a). Our own analysis of six groups of receptors reached a similar conclusion; the binding affinities using high dielectric constants (4 and 20) agreed better with experiment. The difference between calculations using dielectric constants of 4 and 20 was not very apparent except for the case of a highly charged binding pocket in one receptor (Wang C. H. et al., 2016). Aside from the study of higher solute dielectric constants, a residue-dependent dielectric model was also developed for use in an alanine scanning protocol with the MMPBSA method (Simoes et al., 2017). An attempt to modify the solute dielectric environment by incorporating structurally important, explicit water molecules in protein-ligand pockets for MMPBSA calculations was also reported, and it was found to improve the modeling of binding affinities for a series of JNK3 kinase inhibitors (Zhu Y. L. et al., 2014).

A hybrid QM/MM solute was also used in MMPBSA applications for predicting the binding affinities of FabI inhibitors (Su et al., 2015). The study suggested that the prediction results are sensitive to radii sets, GB methods, QM Hamiltonians, sampling protocols, and simulation length. The finding here appears to contradict our prior discussion. This is because the solute dielectric constant is set to 1 (vaccum) due to the explicit consideration of polarization in QM/MM studies. In the study of (Wang C. H. et al., 2016), a high solute dielectric constant was used to mimic polarization implicitly. In general, the use of high dielectric constants washes out the sensitivity to radii and surface definitions.

### The non-polar solvation term

The non-polar (non-electrostatic) solvation free energy contribution (ΔG_non-*polar*_) to the solvation free energy change (ΔG_bind, solv_) arises from the solute cavity formation within the solvent and van der Waals interactions between the solute and the solvent around the cavity (Weeks et al., 1971; Smith and Tanford, 1973; Pratt and Chandler, 1977, 1980; Widom, 1982; Kang et al., 1987; Floris and Tomasi, 1989; Floris et al., 1991; Ashbaugh et al., 1999; Hummer, 1999; Lum et al., 1999; Gallicchio et al., 2000, 2002; Levy et al., 2003; Zacharias, 2003; Gallicchio and Levy, 2004; Su and Gallicchio, 2004; Dzubiella et al., 2006; Wagoner and Baker, 2006; Tan et al., 2007). Until recent times, the non-polar solvation free energy has been simply estimated to be proportional to the solvent accessible surface area (SASA) of the solute:

(9) $$\Delta {\text{G}}_{\text{non - polar}}=\gamma^{*}\text{SASA }+\text{ b}.$$

This is referred to as the classical approach below. The surface tension γ and the correction term b are usually set to be constant for all solute molecules; for example, these are 0.00542 kcal/mol-Å^2^ and 0.92 kcal/mol, respectively, in the AMBER package (Case et al., 2016).

In more modern approaches (Tan et al., 2007), the cavity formation free energy and van der Waals (dispersion) free energy are modeled as separate terms because they scale differently vs. solute size. One way to correlate the cavity formation free energy is to use the volume (SAV) enclosed by the solvent accessible surface. A solvent accessible volume integration, or a solvent accessible surface integration, can be used to compute the dispersion term (ΔG_dispersion_), so the total non-polar solvation free energy can be estimated as:

(10) $$\begin{array}{l} \Delta {\text{G}}_{\text{non - polar}}=\Delta {\text{G}}_{\text{dispersion}}+\Delta {\text{G}}_{\text{cavity}}\\ \;=\;\Delta {\text{G}}_{\text{dispersion}}+\gamma^{*}\text{SAV }+\text{ b}. \end{array}$$

These scaling factors apparently depend on the choices of atomic and solvent probe radii, for example, they are set to 0.0378 kcal/mol-Å^2^ and −0.569 kcal/mol in the AMBER package (Case et al., 2016). It is interesting to note that the coefficient is much higher compared to the classical model since the dispersion term in solvation is always a negative term. The overall non-polar solvation free energies are similar between the classical and modern models, at least for small molecules where all atoms are exposed. The performance of both the classical and modern non-polar solvation models was analyzed, and it was found that the modern approach reduced the root-mean-square deviations of computed binding affinities (both relative and absolute) from experimental values while the correlations changed little from those computed using the classical approach (Wang C. H. et al., 2016).

### The entropy term

The configurational entropy (S) in Equation (1) is often approximated by normal mode or quasi-harmonic analysis. Many proposed methods exist to calculate entropy (Kassem et al., 2015), but it is notoriously difficult to obtain a converged quantity. Thus, further approximations are often utilized; for example, usually only residues within a small sphere (radius of 8–12 Å) centered at the ligand and a limited number of snapshots (<100) are used for normal mode analysis. Furthermore, the single trajectory approach (i.e., that for the complex) is most often used in MMPBSA studies that do not consider any binding-induced structural changes. In such an approach, configurational entropy computed by the normal mode analysis is often omitted completely in the ranking of relative binding affinities as its inclusion often does not improve the agreement with experiment (Yang et al., 2011).

A few new ideas to approximate entropy were reported recently. For example, a new method termed BEERT (Binding Entropy Estimation of Rotation and Translation) was proposed to approximate configurational entropy changes in terms of the reduction in translational and rotational freedom of the ligand upon protein-ligand binding, starting from the flexible molecule approach (Ben-Shalornit et al., 2017). An interaction entropy (IE) method was also proposed to investigate the entropy change upon binding (Duan L. et al., 2016; Duan et al., 2017). The interaction entropy (IE) contribution to binding free energy was defined as

(11) $$-T\Delta S=k_{B}T\ln\langle e^{\beta E_{pl}^{int}}\rangle ,$$

where $\Delta E_{pl}^{int}$ is the fluctuation of the protein-ligand interaction energy for both electrostatic and van der Waals interactions. The ensemble average of $\langle e^{\beta \Delta E_{pl}^{int}}\rangle$ can be extracted from MD simulations, avoiding normal mode calculations. Both developers of BEERT and IE claim that these methods are highly efficient.

### Extension to membrane proteins

The development of implicit membrane models based on existing continuum solvent approaches has advanced, making it possible to extend the MMPBSA method to biological membrane systems. The presence of an implicit membrane adds a complication to the numerical solution of the underlying PBE that is brought about by dielectric inhomogeneities that appear on the boundary surfaces of the computation grid. This issue can be alleviated by employing the periodic boundary condition, which is a common practice for electrostatic computations in MD simulations. The conjugate gradient and successive over-relaxation methods can be adjusted to take into account periodic calculations, but the convergence rate using either method is quite low. This limits their application to MMPBSA calculations which require that a large number of conformations be processed. To improve the convergence rate for use in biomolecular applications, the Incomplete Cholesky preconditioning method and the geometric multigrid method were both extended to incorporate periodicity (Botello-Smith and Luo, 2015). Applications to protein-ligand binding utilizing the newly developed membrane MMPBSA method were also reported (Greene et al., 2016; Xiao et al., 2017).

### Extension to the high-speed screening of ligands

The MMPBSA method has also been utilized as a rescoring method for docking applications. In protein-protein docking, it was found that MMPBSA rescoring was more capable at distinguishing correct complex structures from decoys than ZDOCK scoring in a test set of 46 protein-protein complexes for certain combinations of force fields, solvation models, and dielectric constants (Chen et al., 2016). A new binding affinity estimator, PBSA_E, that is based on MMPBSA inputs, was also optimized for protein-ligand binding. The optimization made use of a training set consisting of high-quality experimental data that was gathered from 145 complex structures. When the predicted binding affinities using PBSA_E were compared with the predicted binding affinities using other popular scoring functions such as GlideXP, GlideSP, and SYBYL_F, the PBSA_E method exhibited improved accuracy in terms of both achieving higher correlations with measured binding affinities and lower root-mean-square deviations (Liu X. et al., 2016). A study on MMPBSA in hierarchical virtual screening (HVS) was reported. This study demonstrated the predictability and validity of using the MMPBSA method for lead discovery as it identified novel inhibitors of the p38 MAP kinase by employing a physics-based scoring function combined with a knowledge-based structural filter (Cao et al., 2014).

A study on the performance of MMPBSA in docking applications found that it depends on the choice of the solvent models among many factors that were analyzed. Specifically, the authors found that the choice of solvent models plays a minor role for one-protein-family/one-ligand cases which represent the unbiased protein–ligand complex sampling. However, for the total dataset with biased sampling, where some proteins and their homologs have an overabundant presence in the dataset, they found that numerical PB solvent methods do not perform as well as GB solvent methods. In addition, they showed that numerical PB methods were more sensitive to whether MD simulations were used for averaging. Such methods may be currently more suitable for individual protein binding free energy rankings where MD simulations can be easily conducted. This study also demonstrated that the numerical noise from screening applications that utilize only one or a few structures should be addressed in future developments of the dielectric model for numerical PB methods (Sun et al., 2014b).

### Extension to other high-performance analyses

MMPBSA was also adapted for high-performance mutational analysis. Single Amino Acid Mutation based change in Binding free Energy (SAAMBE) was developed to predict changes in binding free energy that are brought about by point mutations. SAAMBE utilized 3D structures of protein-protein complexes in a sequence- and structure-based approach. The method was centered around two components: a MMPBSA-based component, and a set of statistical terms obtained from the physico-chemical properties of protein complexes. A better agreement with experimentally determined binding free energy changes over a set of 1,300 mutations in 43 proteins indicated a significant improvement for predictions made using SAAMBE (Li M. H. et al., 2014; Petukh et al., 2015).

Additionally, MMPBSA was adapted into a novel structure-based multiscale approach to identify the key specificity determining residues (SDRs) of PDZ domains that appeared in explicit solvent MD simulations on PDZ-peptide complexes. SDRs were then used together with knowledge-based scoring functions in a proteome-wide search to locate their interaction partners (Tiwari and Mohanty, 2014).

### New toolkits

Over the past few years, several new toolkits were released to facilitate the use of MMPBSA calculations. A free energy workflow tool, FEW, was developed for AMBER to assist in the setup of molecular dynamics simulations in explicit membrane environments. FEW also assists in the setup and execution of effective binding free energy calculations for a single-trajectory implicit solvent/implicit membrane MMPBSA approach that involves multiple ligands binding to the same membrane protein (Homeyer and Gohlke, 2015). g_mmpbsa was developed for GROMACS, and it implemented the MMPBSA approach using subroutines written in-house or sourced from the GROMACS and APBS packages (Kumari et al., 2014). GMXPBSA is another user-friendly suite of Bash/Perl scripts for streamlining MMPBSA calculations for GROMACS users (Paissoni et al., 2014). An easy-to-use pipeline tool named Calculation of Free Energy (CaFE) was published to facilitate both MMPBSA and LIE calculations. CaFe is capable of handling numerous static structure and molecular dynamics trajectory file formats generated by different molecular simulation packages, and it also supports various force field parameters (Liu and Hou, 2016).

## Applications of MMPBSA

### Protein-ligand binding interactions

Since the last comprehensive review of MMPBSA applications in 2015 (Genheden and Ryde, 2015), MMPBSA has continued to see its role in pharmaceutical research and development increase. MMPBSA was widely applied in the development of anticancer compounds where kinases were identified as the most promising targets. Recent inhibitor design includes the epidermal growth factor receptor kinase domain (Li et al., 2015; Moonrin et al., 2015; Zhao et al., 2017), anaplastic lymphoma kinase (Kong et al., 2015), cyclin-dependent kinases (Li X. L. et al., 2014; Czelen, 2016; Dong et al., 2016a,b, 2017; Arba et al., 2017), extracellular signal-regulated kinase 2 (Chen, 2017), casein kinase 2 (Wang X. W. et al., 2014), sphingosine kinases (Fang et al., 2016), Src/Abl tyrosine kinases (Fong, 2015; Ma et al., 2015), RET tyrosine kinase (Gao et al., 2015), PRK1 (Slynko et al., 2014), Akt kinase (Lu et al., 2015), phosphatidylinositol 3 kinase (Bian et al., 2014), and Myt1 kinase (Wichapong et al., 2014). As an illustration of the method's performance, Slynko et al. observed a correlation coefficient of 0.78 between the experimental pIC_50_ of 26 PRK1 inhibitors and computational MMPBSA binding affinities. They also utilized a quantitative structure activity relationship (QSAR) model to improve the correlation to 0.88, combining affinities from MMPBSA, QM/MMGBSA, and Glide scoring (Slynko et al., 2014). There were other attractive targets including indoleamine 2,3-dioxygenase 1 (Zou et al., 2017), translationally controlled tumor protein (Kumar R. et al., 2017), estrogen receptor (Anbarasu and Jayanthi, 2017), MutT homolog 1 (Zhou et al., 2016), survivin (Sarvagalla et al., 2016), CD44 (Nguyen et al., 2016), calmodulin (Gonzalez-Andrade et al., 2016), androgen receptor (Liu H. L. et al., 2016), human topoisomerase I (Guruge et al., 2016), Mcl-1 (Zhao et al., 2015), vascular endothelial growth factor receptor-2 (Wu et al., 2015), tubulin (Liao et al., 2014a,b; Santoshi and Naik, 2014; Suri and Naik, 2015; Suri et al., 2015), the Hsp70 protein family (Bhattacharjee et al., 2015; Schneider et al., 2016), the Hsp90 protein family (Arba et al., 2015), glucose 6-phosphate dehydrogenase (Obiol-Pardo et al., 2014; Zhao et al., 2014), lysozyme (Zhan et al., 2015), p53 (Verma S. et al., 2016), wheat germ agglutinin (Parasuraman et al., 2014), bromodomains (Muvva et al., 2014), matrix metalloproteinases (Zhou et al., 2014), protein arginine methyltransferases (Hong et al., 2014; Yan et al., 2014), human arsenic methyltransferase (Abro and Azam, 2016), Atox1 proteins (Wang X. L. et al., 2014), tyrosyl-DNA phosphodiesterase 2 (Kossmann et al., 2016), and urokinase-type plasminogen activator (Sa et al., 2014).

Applications in the development of antibacterial, antiviral, and antiparasitic drugs are also common, including inhibitor designs to targets such as enoyl reductase (Kamsri et al., 2014; Yang et al., 2017), succinate-ubiquinone oxidoreductase (Zhu X. L. et al., 2014; Xiong et al., 2015), thymidylate kinase (Biswas et al., 2017), peptidoglycan recognition proteins (Sahoo et al., 2014a), *Mycobacterium tuberculosis* pantothenate synthetase (Ntie-Kang et al., 2014), glutamine synthetase (Moreira et al., 2016), FtsZ protein (Zhang H. et al., 2015), and the cytochrome bc1 complex (Zhu et al., 2015). Other efforts focused on HIV viruses, including HIV-1 protease (Li D. et al., 2014; Tzoupis et al., 2014; Chen J. Z. et al., 2015; Meher and Wang, 2015; Chen, 2016; Hu et al., 2016; Sroczynski et al., 2016; Xanthopoulos et al., 2016), gp120 (Wang J. H. et al., 2015), gp41 (Song et al., 2014), reverse transcriptase (Bernardo and Silva, 2014), HIV integrase (Quevedo et al., 2014; Han et al., 2016), and a comparative analysis of inhibitors for HIV-1 and HIV-2 proteases (Chen et al., 2014a). Wright et al. studied 9 inhibitor-bound HIV-1 proteases and compared the absolute binding free energies computed via MMPBSA and MMGBSA with and without normal mode-derived configurational entropy. They found only the values of MMPBSA using the normal mode method were close to experimental values (Wright et al., 2014). Influenza was also an important research topic in antiviral compounds, with targets such as neuraminidase protein (Wang and Chen, 2014; Tran et al., 2015; Chintakrindi et al., 2016; Yang et al., 2016), non-structural proteins (Ai et al., 2014), H7N9 neuraminidase (Phanich et al., 2016), and the M2 proton channel (Homeyer et al., 2016). Other antiviral targets were also examined, such as RNA-dependent RNA polymerase (Yu et al., 2014; Wang J. H. et al., 2016), NS3/4A hepatitis C virus protease (Xue et al., 2014; Fu and Wei, 2015; Meeprasert et al., 2016), human furin (Omotuyi, 2015), and the capsomere of virus-like particles (Li Y. Y. et al., 2014). The method was additionally applied to the identification of novel protease targets for antiviral compounds (Pethe et al., 2017). Studies of antimalarials were also reported. For example, the reduced effectiveness of pyrimethamine, and its relationship to mutation in *Plasmodium falciparum* dihydrofolate reductase, was studied (Mokmak et al., 2014; Abbat et al., 2015). Falcipain-2, a papain family cysteine protease, was also examined as an antimalarial drug target (Omotuyi, 2014).

A third common group of applications are for studies of targets in neural disorders. Targets such as dopamine D2 receptor (Salmas et al., 2017), monoamine oxidase enzymes (Marsavelski and Vianello, 2017), and opioid G protein-coupled receptors (Leonis et al., 2014) were analyzed as antipsychotic compounds. Here, Leonis et al. employed the recently reported crystal structure of the human κ-opioid receptor (κ-OR) to explain the binding mechanism with its antagonist JDTic and agonist SalA. Both JDTic and SalA show that they are capable of forming a favorable complex in the MMPBSA analysis, which was later confirmed by experiment (Leonis et al., 2014). For Parkinson's disease, ligand-binding to the SUR1 receptor (Santos et al., 2016) and the adenosine receptor (Zhang L. H. et al., 2014) was analyzed. Many targets were studied for Alzheimer's disease including β-secretase (Koukoulitsa et al., 2016), angiotensin-converting enzyme (Bhavaraju et al., 2016), acetylcholinesterase and butyrylcholinesterase (Kurt et al., 2017), and inhibitors directly targeting amyloid aggregation (Berhanu and Masunov, 2015). Ataxin-2 protein was studied for the treatment of spinocerebellar ataxia (Sinha et al., 2017), superoxide dismutase for amyotrophic lateral sclerosis (Zhuang et al., 2016), and Niemann-Pick type Cl and C2 proteins (Poongavanam et al., 2016) that occur in rare neurodegenerative diseases.

MMPBSA was also widely applied in studies of many other major diseases. For blood disorders, targets included human serum albumin (Roy et al., 2015; Kragh-Hansen et al., 2016; Yu et al., 2016a,b), collagen-binding alpha 2 beta 1 integrin (Zhang and Sun, 2014), and human alpha-thrombin (Duan L. L. et al., 2016). In the latter study, Duan et al. investigated the binding affinity between human alpha-thrombin and ligand L86 by employing a nonpolarizable AMBER force field and the polarized protein-specific charge (PPC) force field. They found that the PPC binding affinity was closer to the experimental value reported by Nantermet et al. (2003). For immune disorders, studies on Janus kinases (Zhang W. et al., 2016; Wang J. L. et al., 2017), interleukin 10 cytokine (Ni et al., 2017), and receptor-related orphan receptor-gamma-t (Wang F. F. et al., 2015), were reported. For inflammatory disorders, targets such as COX-2 (Chaudhary and Aparoy, 2017), interleukin 6 (Verma R. et al., 2016), toll-like receptors (Shen et al., 2016), human leukocyte antigen (Kongkaew et al., 2015), chymase enzyme (Verma et al., 2017), tumor necrosis factor (Ivanisenko et al., 2014), and Nalp3 (Sahoo et al., 2014b) were studied. For diabetes, analyses of targets included phosphorylase kinase (Begum et al., 2015), glycogen synthase kinase (Arfeen et al., 2015), protein kinase C beta II (Grewal and Sobhia, 2014), and dipeptidyl peptidase-4 enzyme (Gu et al., 2014; Sneha and Doss, 2016). In a search for compounds for the treatment of chronic kidney disease, Vitamin D receptor and cytochrome P450 were analyzed (Nagamani et al., 2016). Malonyl-CoA decarboxylase (Ling et al., 2016), sirtuins (Karaman and Sippl, 2015), adipocyte fatty-acid binding protein (Chen et al., 2014b), 11β-hydroxysteroid dehydrogenase type 1 (Qian H. Y. et al., 2016), and protein tyrosine phosphatase 1B (Kocakaya, 2014) were reported for treatments of other metabolic diseases. For other diseases and disorders, targets such as pyrroline-5-carboxylate reductase for cutis laxa (Sang et al., 2017), renin complexes for hypertension (Tzoupis et al., 2015), and the type 1 receptor of TGF beta for wound healing (Gesteira et al., 2017) were examined.

There were a wide range of applications to targets outside the scope of pharmaceutical research. Although these studies are not related to drug discovery, accurate modeling of protein-ligand binding is also important in a wide variety of other contexts. For example, Starovoytov et al. calculated MMPBSA binding affinities of BPA-A, BPA-C, and BPA-D bound to human estrogen-related receptor γ in their toxicology research. Their analysis showed that BPA-A was the strongest binder to the receptor (Starovoytov et al., 2014). Other similar studies include globin, for its catalytic mechanism in the hydrolysis of substituted phenyl hexanoates (Ercan et al., 2014), the role of conserved residues in substrate binding to *Brassica rapa* auxin amidohydrolase (Smolko et al., 2016), the effect of hydrophobic interactions in substrate binding to recombinant enzyme carboxylesterase (Shao et al., 2014), the substrate-enzyme interactions of endo-1,4-β-xylanase (Zhan et al., 2014), azoreductase protein for the biodegradation of azo dyes (Dehghanian et al., 2016; Haghshenas et al., 2016), cytochrome P450 2A6 for nicotine addiction (Lu et al., 2014), Cel48F for producing bioethanol via fiber degradation (Qian M. D. et al., 2016), rubisco for biofuel production (Siqueira et al., 2016), streptavidin-biotin complex in biochemical sensing (Liu F. J. et al., 2016), sorotidine 5-monophosphate decarboxylase for its impressive rate enhancement (Jamshidi et al., 2014), tyrosyl-tRNA synthetases for genetic encoding of unnatural amino acids (Ren et al., 2015), T7 RNA polymerase for generating RNA labels (Borkotoky et al., 2016), AF9 in the YEATS family for the recognition of H3K9ac (Wang Q. et al., 2016), cysteine protease 1 precursor from Zea for the hydrolysate of corn gluten meal (Liu et al., 2014), folate receptor alpha for producing milk with high folate concentration (Sahoo et al., 2014c), and both acid amido synthetase (Wang X. et al., 2015) and brassinosteroid (Lei et al., 2015) for plant growth.

### Protein-protein binding interactions

There are many applications of MMPBSA that involve the calculation of protein-protein or protein-peptide binding affinities. For anti-cancer applications, we saw studies on complexes of an ankyrin repeat with integrin-linked kinase binding to PINCH1 (Gautam et al., 2014, 2015), cyclin-dependent kinase 8 with cyclin C (Xu et al., 2014), Hsp90 with Cdc37-derived peptides (Wang L. et al., 2015), and p53 with MDMX (Shi et al., 2015). As an illustration, Wang et al. designed an eleven-residue peptide that was able to bind to Hsp90 with a predicted affinity of 6.9 mM, which was comparable to the experimental value of 3.0 mM (Wang L. et al., 2015).

For anti-viral applications, research efforts were focused on analyzing interactions for ankyrin and domain III of the envelope protein of dengue virus II (Chong et al., 2015; Dubey et al., 2017), a modular capsomere of a murine polyomavirus (MPV) VLP designed to protect against influenza (Zhang L. et al., 2014), antibody recognition of immunoglobulin 2D1 for influenza virus H1N1 (Leong et al., 2015), differential structural dynamics and antigenicity of two HA-specific CTL epitopes binding to HLA-A^*^0201 for influenza virus H5N1 (Sun and Liu, 2015), a complex of histone deacetylase 6 and ubiquitin-specific protease 5 for influenza virus A (Passos et al., 2016), and for a complex of an antibody and epitope variant of HIV-1 p24 capsid protein (Karim et al., 2015). For antibacterial applications, there were studies of salamander PGRP1 with its splice variant adPGRP1a for the innate immune system (Qi Z. T. et al., 2017), and an adsorption mechanism of human beta-defensin-3 on bacterial membranes (Lee et al., 2016). Based on their MMPBSA analysis, Lee et al. found that the binding affinity of human beta-defensin-3 bound to a gram-positive membrane is over 3 times higher than when it is bound to a gram-negative membrane. Interestingly, this difference is mostly derived from electrostatic interactions, consistent with a net charge that is three times larger for gram-positive membranes compared to gram-negative membranes (Lee et al., 2016).

Studies of protein-protein interactions were also found for other diseases and disorders including serum paraoxonase 1 with high-density lipoprotein for antiatherosclerotic activity (Patra et al., 2014), vascular endothelial growth factor A with binding domains of anti-angiogenic agents for retinal neovascular degenerative diseases (Platania et al., 2015), angiotensin-converting enzyme with Angiotensin-II for hypertension (Guan et al., 2016), titin with T-cap/telethonin for dilated cardiomyopathy (Kumar D. T. et al., 2017), CC chemokine ligand 5 (CCL5) with human neutrophil peptide-1 (HNP1) for chronic inflammatory diseases (Wichapong et al., 2016), and ANKS6 with ANKS3 for polycystic kidney disease (Kan et al., 2016). In the study of CCL5 binding with human HNP1, Wichapong et al. reported a correlation coefficient of *r* = 0.66 between experiment and MMPBSA results for different species of CCL5. In addition, they confirmed that the entropy change upon binding was negligible in this study, so the entropy term could be ignored when a relative binding free energy was considered (Wichapong et al., 2016).

In addition, there were research efforts that focused on basic mechanisms such as protein stability and conformational dynamics (Bhavaraju and Hansmann, 2015; Getov et al., 2016). Many other studies were also reported, including an analysis of full length amylin oligomer aggregation (Berhanu and Masunov, 2014), effects of single point mutations on amyloid formation (Bhavaraju and Hansmann, 2015; Getov et al., 2016; Petukh et al., 2016), interactions between methylated histone H3 and effector domains of the PHD family in pursuit of a molecular mechanism of epigenetics (Grauffel et al., 2015), the binding mechanism of actin-depolymerizing factor 1 and G-actin (Du et al., 2016), the interaction between phosphotyrosine binding domains and peptides for neuronal development, immune responses, tissue homeostasis, and cell growth (Sain et al., 2016), the cognate transducer complex srII-htrII for the downstream signaling mechanism of sensory rhodopsin (Sahoo and Fujiwara, 2017), the binding of CBP to c-Myb for understanding the exact function of CBP and its interaction with c-Myb (Odoux et al., 2016), the catalytic stability of the tetrameric complex of cystathionine gamma-lyase (El-Sayed et al., 2015), the study of the mechanism of a molecular chaperone using acid-stress chaperone HdeA and its substrate protein (Zhou et al., 2017), and the binding of thiopeptide to a ribosomal subunit in order to understand the structure–activity relationship of thiostrepton derivatives (Wolf et al., 2014).

There are also several non-biomedical applications such as a study on the interactions between two adjacent gamma tubulins within the gamma-tubulin ring complex for growing yeast (Suri et al., 2014), between beta-sheet regions in corneous beta proteins of sauropsids to explain its stability and polymerization into filaments (Calvaresi et al., 2016), and the recognition of Avr protein by eukaryotic transcription factor xa5 of rice to understand the gene-for-gene mechanism that governs the direct interaction of R-Avr protein (Dehury et al., 2015). In the study by Suri et al. computational alanine scanning was employed to determine hotspot contributions in the interaction between gamma tubulins. Their analysis showed that most hotspot mutations reduce affinity by 1.16 kcal/mol, while for very crucial amino acids, such as Asp252 and Arg341, the affinity was decreased by more than 10 kcal/mol, which correlated well with experiment (Suri et al., 2014).

### Complexes involving nucleic acids

For DNA-protein interactions, there were mechanistic studies involving proteins such as HU, one of the major nucleoid-associated proteins for stabilizing DNA bending (Kim et al., 2014), highly conserved chromatin protein Cren7 for cellular processes such as transcription, replication, and repair (Chen L. et al., 2015), MutS, which recognizes mismatched DNA in DNA repair using ATP (Ishida and Matsumoto, 2016), copper nucleases for predicting electrostatic interactions with B-DNA (Liu C. M. et al., 2016), and metallopeptides binding to the Drew-Dickerson dodecamer (Galindo-Murillo and Cheatham, 2014). For RNA-protein interactions, the binding of toll-like receptor 3 and 22 was studied in relation to fish viral diseases (Sahoo et al., 2015), and the RNA-recognition motif of RNA-binding protein was analyzed for anti-cancer actitivies (Chang et al., 2016). Two studies on DNA-DNA interactions were also reported, including the calculation of binding affinities of short double stranded oligonucleotides (Yesudas et al., 2015), of three-quartet intramolecular human telomeric DNA G-quadruplexes (Islam et al., 2016), and of strand-strand interactions in a human telomeric tetrameric quadruplex (Chaubey et al., 2015). Yesudas et al. studied oligonucleotides (9–20 mers DNA) with the “3-trajectory” approach, without counter ions, by using GBSA, PBSA, and 3D-RISM-KH methods to calculate binding free energies. They showed that the 3D-RISM-KH method was in better agreement with the experimental data for larger oligonucleotides while the GBSA method performed better for smaller oligonucleotides (Yesudas et al., 2015).

There were also several studies involving small ligands binding to nucleic acids. Interactions of anticancer compounds with DNA, such as cisplatin and oxaliplatin (Jalili et al., 2016), distamycin with the DNA minor groove (Jalili and Maddah, 2017), and plant alkaloid chelerythrine with the human telomere sequence (Ghosh et al., 2015) were reported. Aminobenzimidazole binding to an internal ribosome entry site was studied for its anti-hepatitis C virus effect (Henriksen et al., 2014). The interactions of histone-derived antimicrobial peptides buforin II and DesHDAP1 with DNA were also investigated (Sim et al., 2017). Basic mechanistic studies were carried out, including cationic porphyrin-anthraquinone hybrids binding to DNA duplexes (Arba and Tjahjono, 2015) and G-quadruplexes (Arba et al., 2016), as well as ligands binding to riboswitches upon mutation (Hu et al., 2017). The interactions of reactive metabolites of anticancer compounds with DNA were also analyzed (Tumbi et al., 2014). In the binding of ligands to riboswitches, Hu et al. analyzed the relative binding free energies for a guanine riboswitch (GR) and a GUA complex relative to three complexes: 6GU (3.4 kcal/mol), 2BP (5.48 kcal/mol), and XAN (6.19 kcal/mol). These values were in good agreement with experimental observations of 3.21, 4.12, and 5.47 kcal/mol, respectively (Hu et al., 2017).

### Guest-host and nano systems

For guest-host systems, several studies were reported. Two octa acid hosts complexed with six guest molecules were analyzed by Bhakat and Soderhjelm for resolving the problem of trapped water in binding cavities. They performed a well-tempered funnel metadynamics (WT-FM) and MMPBSA analyses for the two octa-acid hosts, OAH (without methyl groups) and OAMe (with methyl groups), using both GAFF and OPLS force fields. Their analyses showed that the binding affinities of WT-FM are basically similar to experimental values while MMPBSA results have errors in the range of 5–10 kcal/mol due to its approximate nature (Bhakat and Soderhjelm, 2017). A binding interaction was analyzed to show that electrostatic interactions have the largest contribution to the stability of the cucurbituril-pseudorotaxane complex (Malhis et al., 2015). Complex stability was analyzed for multiple systems, such as the 1:1 and 1:2 inclusion complexes formed by nor-*Seco*-cucurbit[10]uril and 1-adamantanmethylammonium in water (El-Barghouthi et al., 2015), cyclodextrin-Ibuprofen complexes (Wang R. M. et al., 2015), E-selectin-oligosaccharide complexes (Barra et al., 2017), and the naringenin-2,6-dimethyl β-cyclodextrin inclusion complex (Sangpheak et al., 2014). Finally, enantiomeric discrimination of chiral organic salts by chiral aza-15-crown-5 ether with C1 symmetry was reported (Kocakaya et al., 2015).

For nano systems, a mechanism explaining how C-60 can block potassium ion channels was proposed (Calvaresi et al., 2015). The authors showed that a new binding site for C-60 exists in the channel cavity at the intracellular entrance of the selectivity filter. The escape barrier from the binding site is ~21 kcal/mol as calculated via the umbrella sampling method, in good agreement with the MMPBSA result. MMPBSA was also used in the theoretical design of the cyclic lipopeptide nanotube as a molecular channel in the lipid bilayer (Izadyar et al., 2015; Khavani et al., 2015, 2017), in the study of the enzyme immobilization mechanism of alpha-chymotrypsin onto carbon nanotubes in organic media (Zhang L. Y. et al., 2015), and the mechanism of carbon nanotube activation of subtilisin Carlsberg in polar and non-polar organic media (Zhang L. Y. et al., 2016).

### Monomer stability

For single biomolecule stability research, we saw reports on the structures and energies of the alternate frame folding calbindin-D_9k_ protein (Tong et al., 2015), the effect of copper ions in the stability and structural change of human growth protein (Tazikeh-Lemeski, 2016), and the role of potassium in stabilizing the human telomeric intra-molecular G-quadruplex structure (Wang Z. et al., 2015; Wang and Liu, 2017). In their study of calbindin-D_9k_, Tong et al. found two transition states and an intermediate state with a first rate-controlling barrier of 4.7 kcal/mol and a second barrier of 1.7 kcal/mol using MMPBSA, both of which are in good agreement with experiment (Tong et al., 2015).

## Current limitations and future directions

MMPBSA methods are widely applied to calculate binding affinities at a reasonable computational cost. These computational analyses have provided a large volume of valuable predictive results in a wide variety of studies. Even though MMPBSA is known to be less accurate than some of the more computationally expensive methods, like the free energy perturbation and thermodynamic integration methods, the qualitative agreement is often good enough to aid collaborative efforts involving both computational and experimental researchers. Developers are also actively working to improve MMPBSA methods for higher accuracy and efficiency by introducing better solute and solvent models, by porting the expensive energy computation (mostly involving the solvation terms) to faster GPU platforms, and by improving entropy estimations. Efforts are also needed to extend the MMPBSA method for various screening purposes that involve a large number of ligands and/or mutations to achieve a higher overall level of accuracy and efficiency. It is apparent that applications of the MMPBSA method have grown considerably in many different areas of biomolecular study. Most of these applications involve protein-ligand binding affinity calculations due to their utility in drug discovery research efforts. There are also many applications in the study of biomacromolecular complexes. It is also noteworthy that a few guest-host and nano systems utilized MMPBSA calculations, indicating a wider development space for this method in the future.

## Author contributions

CW: Did the literature research and drafted the review; DG: Extensively revised the review; LX: Wrote the membrane protein extension review; RQ: Wrote the solvation term review; RL: Designed the overall structure of the review and was involved in drafting the review. All people polished the final version.

### Conflict of interest statement

The authors declare that the research was conducted in the absence of any commercial or financial relationships that could be construed as a potential conflict of interest. The reviewer WC and handling Editor declared their shared affiliation.
